# Dysphagia and gastrointestinal disorders in Parkinson’s disease: towards a diagnostic standard operating procedure for the interprofessional neurological outpatient care team

**DOI:** 10.1007/s00702-026-03195-z

**Published:** 2026-08-21

**Authors:** Inga Claus, Carsten Buhmann, Alessandra Fanciulli, Stephan Klebe, Franz Marxreiter, Patrick Süß, David Weise, Tobias Warnecke

**Affiliations:** 1 Department of Neurology, University Hospital of Muenster, Albert-Schweitzer- Campus 1, 48149 Muenster, Germany; 2 Department of Neurology, University Medical Center Hamburg-Eppendorf, Martinistraße 52, 20246 Hamburg, Germany; 3 Department of Neurology, Medical University of Innsbruck, Innsbruck, Austria; 4 Department of Neurology, Knappschaft Hospital Recklinghausen, Recklinghausen, Germany; 5 Center for Movement Disorders, Passauer Wolf, Bad Gögging, Neustadt an der Donau, Germany; 6 Department of Molecular Neurology, University Hospital Erlangen, Friedrich- Alexander-Universität Erlangen-Nürnberg, Erlangen, Germany; 7 Department of Neurology and Pain Therapy, Asklepios Fachklinikum Stadtroda, Stadroda, Germany; 8 Department of Neurology, University of Leipzig, Leipzig, Germany; 9 Department of Neurology and Neurorehabilitation, Klinikum Osnabrueck, Osnabrueck, Germany

**Keywords:** Parkinson’s disease, SOP, Dysphagia, Gastrointestinal disorders

## Abstract

Gastrointestinal Disorders in people with Parkinson’s disease (PwP) are able to cause widespread clinical symptoms which can be challenging to classify in day-to-day clinical practice. In the upper gastrointestinal tract (GIT) oropharyngeal dysphagia, esophageal motility disorders as well as delayed gastric emptying may cause nutrition problems and reduce the efficacy of oral pharmacotherapy. In the lower GIT, bacterial overgrowth and prolonged colorectal transit times induce different stages of constipation leading to reduced quality of life and GIT comorbidities as well. Most of these symptoms reflect alpha-synuclein pathology in both the enteric nervous system and at various sites of the peripheral and central nervous systems. Besides, the growing use of various device aided therapies may additionally raise specific clinical questions about the interference with the GIT motility. This standard operating procedure (SOP) is intended to provide specific recommendations for diagnostic steps as well as therapeutic options, especially for interprofessional neurological outpatient care teams.

## Introduction

There is large evidence for (Agachan et al. 1996) motility disorders of the gastroininal tract (GIT) to cause widespread clinical symptoms including dysphagia, bloating, nausea, vomiting, gastroparesis and constipation in most patients suffering from Parkinson’s disease (PD) (Suttrup and Warnecke 2016; Warnecke et al. 2022). GIT symptoms, especially constipation, but also dysphagia, gastroparesis, and irritable bowel syndrome without diarrhoea might occur even in prodromal stages of the disease, and increase during disease duration (Cersósimo et al. 2013; Fasano et al. 2025; Konings et al. 2023), a process linked to the progression of the underlying α-synuclein pathology. Recent studies suggest a body-first subtype of PD where pathology starts in the peripheral, most likely the enteric nervous system, leading to a bottom-up invasion of alpha-synuclein pathology via autonomic neurons to the sympathetic trunk, brainstem and finally cortical brain structures (Horsager et al. 2020). As a result, early accumulation of phosphorylated alpha-syn aggregates in the enteric and central nervous system leads to impairment of oral, pharyngeal and esophageal phases of swallowing as well as disturbed gastric motility and colon peristalsis, altered ininal permeability and constipation caused by additional autonomic dysfunction of the colon (Warnecke et al. 2022; Beach et al. 2020; Braak et al. 2003; Leta et al. 2023; Spencer and Hu 2020). Various studies showed alpha-synuclein deposition in the myenteric and submucosal plexuses and mucosal nerve fibers with its distribution associated with gastroininal symptoms along the entire GIT (Warnecke et al. 2022; Fasano et al. 2025; Braak et al. 2003). These alterations might lead to severe complications like malnutrition, dehydration, delayed or insufficient medication effects and in worst cases aspiration pneumonia, which is still the leading cause of death in affected patients (Warnecke et al. 2022). Delayed gastric emptying, intestinal obstruction and complications like megacolon might result in need for intensive medical treatment. Addressing these issues in (primary care and) outpatient neurological care of People with Parkinson’s disease (PwP) is therefore of vital importance.

In addition to the gastroininal symptoms themselves, impaired absorption of dopaminergic medication also results in clinical challenges. There is growing evidence for different aspects of (Araki et al. 2009) GIT transport barrier dysfunction leading to altered levodopa pharmacokinetics, i.e. reduced levodopa response and symptom fluctuations (Leta et al. 2023). The presence of dysphagia delayed gastric emptying (DGE), moderate to severe constipation but also disruptions of gut microbiota, i.e. Helicobacter pylori infection, small ininal bacterial overgrowth, as well as special food-drug interactions may result in exacerbation of motor PD symptoms (Leta et al. 2023).

## Scope

The present manuscript presents a practice-orientated, detailed standard operating procedure (SOP) for general neurologists as well as other members of interprofessional neurological outpatient care teams for PwP (i.e. Parkinson Nurses, Parkinson assistants (PASS) as well as interprofessional network teams), but might also be useful for trained Movement Disorder specialist teams as well to differentiate between GIT associated motility disorders and transport barrier dysfunction to ask the right questions during medical history taking, to initiate or arrange appropriate diagnostic examinations and to provide sufficient therapy options depending on individual needs and challenges (Prell et al. 2020). It aims to raise awareness of PD-associated GIT symptoms in general clinical routine and to describe standardized procedures for the detection, diagnostic evaluation, treatment, and follow-up of dysphagia, as well as other gastrointestinal impairment in PwP. All specific GIT disorder patterns are addressed separately. This manuscript and the containing suggestions are the results of a workshop which took place on the 26. Expert Meeting Parkinson from 28.-29.11.2025 in Frankfurt/Main Germany. Opinion leaders from German-speaking countries discussed the problem of dysphagia and GIT disorders in PD in detail as part of this special workshop which was led by a Parkinson’s expert with special dysphagia expertise, who is also the last author from the outcome publication. One specific GIT topic was assigned to each expert performing individual Pubmed research. Afterwards, all aspects and suggestions given in this manuscript have been discussed carefully among all 8 authors with modification of recommendations if not all PD experts agreed.

## Background and definitions

As in PD the whole GIT can be affected, Table 1 provides an overview of different GIT levels (oropharyngeal, esophageal, gastrojejunal, colorectal) explaining level-related possible motility disorders as well as typical locations of associated medication transport dysfunction. Each level of possible GIT impairment should be kept in mind during screening examination.

**Table 1:** Description of most relevant GIT impairment patterns on different levels

GIT Level GIT impairment pattern	
Oropharyngeal Motility disorders Medication transport disorders	Insufficient pharyngeal bolus clearing (Warnecke et al. 2022) Residues located predominately in the valleculae > sinus piriformes (Warnecke et al. 2022) Penetration/aspiration events with increasing risk for silent aspiration in later stages of the disease (Warnecke et al. 2022) Delayed swallowing reflex (Warnecke et al. 2022) Oropharyngeal Freezing (Labeit et al. 2020) -Festination -Trembling -Akinesia Oropharyngeal residue of medication (Leta et al. 2023)
Esophageal Motility disorders Medication transport disorders	Esophageal motility disorders (Sung et al. 2010; Suttrup et al. 2017) -Hypomotility -Spasms Impaired opening of upper and lower esophageal sphincter (Suttrup et al. 2017) Esophageal spasms with insufficient medication clearing (Leta et al. 2023)
*Gastrojejunal* Motility disorders Medication transport disorders	Delayed gastric emptying (DGE) Defined as > 60% retention at 2 h postprandially and/or > 10% retention at 4 h after ingestion of a radioactive technetium Tc 99 m-labeled solid food (Pasricha and Parkman 2015) Small intestinal bacterial overgrowth (SIBO) Defined as an increased concentration of bacteria above 10^5^ colony-forming units/ml or the presence of unusual colonic-type bacteria in the small inine (Leta et al. 2023) Gastroparesis with delay transport of medication (Fasano et al. 2025; Leta et al. 2023))
*Colorectal* Motility disorders Medication transport disorders	Slow transit constipation caused by impaired colonic motility leading to prolonged transit times (Leta et al. 2023) Outlet constipation caused by anorectal dysfunction (Fasano et al. 2025) Constipation with medication overload (Leta et al. 2023)

The prevalence of any oropharyngeal dysphagia (OD) is estimated up to 82–95%% in PWP (Kalf et al. 2012; Nienstedt et al. 2019; Pflug et al. 2018) with aspiration in about 25% of all PWP (Pflug et al. 2018). Unfortunately, only 20–50% of affected patients are aware of swallowing function disturbance and less than 10% report spontaneously to their neurologists or speech and language therapists (SLPs) as not recognizing swallowing impairment as being a PD symptom (Warnecke et al. 2022; Nienstedt et al. 2019; Pflug et al. 2018; Bird et al. 1994). The range of dysphagia occurrence can spread from prodromal to late stages but usually worsens during disease duration ((Kalf et al. 2012; Patel et al. 2020; Galli et al. 2025). OD might lead to malnutrition, dehydration, aspiration pneumonia und impaired medication intake leading to motor fluctuations as well (Buhmann et al. 2019; Fukae et al. 2020; Hirano et al. 2025). Esophageal motility disorders might occur in prodromal stages as well and can be found in the majority of PwP (Suttrup and Warnecke 2016; Sung et al. 2010). The prevalence of disturbed gastric emptying in PwP ranges from 70 to 100% concerning all disease stages and leads to levodopa pharmacokinetic disturbance, i.e. impaired enteral adsorption and consecutive fluctuations as well as delayed or missing medication effects (Fasano et al. 2025). DGE might also be secondary caused by medical comorbidities (i.e. diabetes mellitus etc.) or worsened by levodopa intake itself by binding gastric dopaminergic receptors in a dose-dependent manner (Leta et al. 2023). SIBO is defined as an increased concentration of bacteria above 10^5^ colony-forming units/ml or the presence of unusual colonic-type bacteria in the small inine. It is suggested as well to negatively influence levodopa absorption with a prevalence of up to 55% in PwP (Beckers et al. 2022). Possible risk factors include GI dysfunction, disease duration and proton pump inhibitor therapy, as well as maybe a Helicobacter pylori infection (Leta et al. 2023). These factors in combination with other covariates are supposed to lead to worsening of motor function in PwP by causing an increased frequency of motor fluctuations as well as prolonged ‘off’ periods in affected patients. Constipation occurs in up to 80% of PwP and is considered to be the most frequent autonomic symptom in PD (Fasano et al. 2025; Jost 2017; Jost 2017; Knudsen et al. 2027), often occurring years before the onset of motor symptoms (Savia et al. 2009; Yao et al. 2023). Concerning constipation in PwPs impaired colonic motility leads to prolonged colonic transit times that become clinically apparent in slow transit constipation (Leta et al. 2023). In addition, anorectal dysfunction with defecator problems leads to ‘outlet’ constipation, occurring in > 60% of PwP (Fasano et al. 2025).

## Screening methods

The use of adequate screening methods is essential for timely and accurate diagnosis as well as for initiating further treatment. Especially PwPs with the “Body-first” subtype should undergo close clinical monitoring from the time of PD diagnosis. An important role plays a targeted medical history which should be applied every 3–6 months, starting with the simple question of which GIT part might show grea impairment. Furthermore, after explaining the association of PD and GIT disorders the PwPs should be asked to point to the most affected area (see picture 1). If necessary in medical context, validated screening tools may help to frame and document the clinical problem (Suttrup and Warnecke 2016).

Table 2 gives an overview of possible useful questions as well as available questionnaires and screening tools.

**Table 2:** Suggested screening methods for PD associated GIT disorders

General introductory questions	
	Do you have difficulties with the GIT?
	Which part is most affected? Please point to the appropriate area.
	How frequently do you have bowel movements?
	Do you have problems with reduced mobility after individual medication intake (question about delayed, partial or no ON phases)?
	Do these problems worsen after meals?
	Has there been any significant weight loss recently?
*Level of GIT impairment* *Oropharyngeal*	
Suggested questions:	
During medical history, PWPs should be asked about	Difficulties in swallowing or medication intake
	Coughing, choking, wet voice
	Severe drooling or sialorrhea
	Relevant loss of weight
	Episodes of unexplained fever
	Pneumonia events within the last month (Suttrup and Warnecke 2016)
Validated questionnaires	SDQ* (Manor et al. 2007)
	MDT-PD (Simons et al. 2014)
	NMSQ; Question 3: “difficulty swallowing food or drink or problems with choking” (Chaudhuri et al. 2006)
	MDS-UPDRS; Question 2.3 of the UPRDS II: “problems swallowing pills or eating meals” (Goetz 2008)
Screening tools	Modified water test (maximum swallowing volume < 20 mL (Kalf et al. 2011)
	Eating Assessment Tool-10 (EAT-10) (Schlickewei et al. 2021)
	Gugging Swallowing Screen (GUSS) (Frank et al. 2021)
*Esophageal*	
Suggested questions	
During anamnesis, PWPs should be asked about	Nausea, vomiting, early satiety, postprandial fullness, loss of weight* (Mukherjee et al. 2016)
	Epigastric pain during swallowing* (PWPs should be asked to show the exact location)
*Gastrojejunal*	
Suggested questions	
During anamnesis, PWPs should be asked about	Nausea, vomiting, early satiety, postprandial fullness* (Mukherjee et al. 2016)
*Colorectal*	
Suggested questions	
During anamnesis, PWPs should be asked about	Frequency of bowel movements (Josh 2010)
	Abdominal pain, anal sphincter function (18), straining during defecation, and a sensation of incomplete evacuation
Validated questionnaires	SCOPA-AUT (Visser et al. 2004)
	GDQ (Knudsen et al. 2027)
	CCS* (Agachan et al. 1996; Knudsen et al. 2027)
	PAC-SYM (Raeder et al. 2023)

SDQ: Swallowing disturbance questionnaire; MDT-PD: Munich dysphagia test – Parkinson’s disease; NMSQ: Non-motor symptoms questionnaire; MDS-UPDRS: Movement Disorder Society – Unified Parkinson’s disease rating scale; SCOPA-AUT: Scales for Outcomes in Parkinson’s disease – Autonomic dysfunction; GDS: gut dysmotility questionnaire; CCS: Cleveland Constipation Scale; PAC-SYM: patient assessment of constipation-symptoms. *Suggested main screening method per GIT level based on the authors’ clinical experience

Regarding to self-reported swallowing difficulties in comparison to instrumental assessment results, PwPs often show a mismatch in occurrence and severity of reported symptoms which might also apply to perception about other GIT level impairment (Logemann et al. 2008). However, from the author’s clinical experience PwPs are often able to address their most impaired GIT level relatively well so these questions should be regarded as a general screening start without any claim to complete reliability.

Concerning the use of validated questionnaires, it should be noted as well that several studies question the reliability of some of these questionnaires for screening purposes in PD. For example, sialorrhea (drooling) does not seem to be a reliable clinical sign for sufficient detection of severe dysphagia with critical aspiration (Nienstedt et al. 2018). The EAT-10 and GUSS questionnaires showed benefits in other diseases, but less so in PD (Schlickewei et al. 2021; Frank et al. 2021). Even PD specific screening tools as questions self-reporting dysphagia in NMS-Quest 3 and UPRDS II.3 or the MDT-PD questionnaire proved to be insufficiently reliable or controversial regarding the results detecting critical aspiration (Buhmann et al. 2019). The Modified Water Swallow (MWST) is useful for general detection of dysphagia with moderate sensitivity and specificity, but in PD the validity is substantially limited especially for detecting aspiration events (Pflug et al. 2019). Concerning constipation, several questionnaires like the Cleveland Constipation Scale (CCS), the gut dysmotility questionnaire (GDS) and the patient assessment of constipation- Symptoms (PAC- SYM), among others, have been evaluated for their use in PD. Yet, they may be less suited in clinical practice, as they may underestimate colonic dysfunction in PD. In example, in comparison to objective measures of colonic dysfunction, constipation was underreported in one study using the CCS (Knudsen et al. 2027).

Therefore, from their clinical experience, the authors would suggest using the SDQ questionnaire for screening for oropharyngeal dysphagia as well as the CCS for constipation evaluation (Agachan et al. 1996; Manor et al. 2007). Concerning esophageal and gastric level impairment, careful exploration of aforementioned symptoms should be performed (see Table 2).

## Diagnostic tools

Although detailed history is mandatory and as mentioned above screening tools applying validated questionnaires - though with some limitations - are helpful in daily practice to address potential GIT disturbance in PwP, both approaches are not able to reliably detect slight or even severe forms of upper and lower GIT impairment (Suttrup and Warnecke 2016).

Therefore, appropriate objective diagnostic instrumental tools should be applied in cases of unclear or severe GIT disturbance (see Table 3). Possible “red flags” might be (Suttrup and Warnecke 2016; Lam et al. 2007; Nóbrega et al. 2008; Schindler et al. 2021):

- Relevant loss of weight.
- Prolonged mealtimes due to swallowing difficulties.
- Coughing while eating.
- Insufficient response to oral medication therapy.
- Clinical signs of aspiration events.
- Severe drooling.

**Table 3:** Diagnostic reliable instrumental tools and clinical indications for upper and lower GIT impairment examination

Diagnostic instrumental tools	Clinical indications
*Oropharyngeal*	
Flexible Endoscopic Evaluation of Swallowing (FEES)	“Gold standard” for detection of oropharyngeal dysphagia and differentiation between specific dysphagia phenotypes/disturbance patterns
	Evaluation of swallowing difficulties for different food consistencies
	(Suttrup and Warnecke 2016), (Kalf et al. 2011), (Langmore 2003)
Videofluoroscopy (VFSS)	“Gold standard” alongside FEES for examination of oral and pharyngeal stages of swallowing including the upper esophageal sphincter
	Detection of dysphagia for several different food and liquid consistencies
	(Suttrup and Warnecke 2016), (Kalf et al. 2011), (Logemann 1998)
*Esophageal*	
High-resolution manometry (HRM)	Detection of combined or isolated esophageal dysphagia
	Evaluation of esophageal impairment even in earliest stages of the disease
	Evaluation of pressure levels in the tubular esophagus as well as opening characteristics of upper and lower esophageal sphincter (Sung et al. 2010; Suttrup et al. 2017)
Esophagogastroduodenoscopy (EGD)	Detection of other causes of ED, i.e. esophagitis or Zencker’s diverticulum (Mukherjee et al. 2016)
*Gastrojejunal*	
Technetium (Tc) 99 m method	Apply of Tc 99 m-labeled solid food
	Measurement of food retention after 2 h and 4 h after ingestion (see definition section) (Pasricha and Parkman 2015)
^13^C-octanoate breath test	Application of a meal containing 13 C-sodium octanoate
	Quantitative measurement via breath tests
	Nonradioactive method (Goetze et al. 2005)
Magnetic resonance imaging (MRI)	Dynamic measurement of gastric emptying, volume changes and motility contractions
	Apply of contrast-enhanced meals is necessary (Unger et al. 2010)
Electogastrography (EGG)	Noninvasive test for recording of stomach’s electrical slow waves
	Evaluation of motility disorders in PwP, even in early stages
	(Araki et al. 2009; Naftali et al. 2005)
Esophagogastroduodenoscopy (EGD)	Detection of other causes of gastroduodenal impairment, i.e. HP infection or SIBO (Mukherjee et al. 2016)
Breath tests (Urea/Lactulose)	Detection of HP or SIBO infection (Leta et al. 2023)
*Colorectal*	
Use of radiopaque markers	Evaluation of Colonic transit time
Wireless motility capsules	Evaluation of Colonic transit time
Anorectal manometry	Evaluation of defecatory disorders
Rectal balloon expulsion	Evaluation of defecatory disorders
Defecography	Evaluation of defecatory disorders
Coloscopy	Detection of other causes of constipation (Mukherjee et al. 2016)

HP: Helicobacter pylori, SIBO: Small intestine bacterial overgrowth

## Treatment options

The appropriate selection and correct application of adequate therapeutic options tailored to each patient’s individual impairment patterns represent an essential component of PD management (Warnecke et al. 2022). In some cases, the efficacy of selected methods should be confirmed by applying instrumental examination tools as outlined in detail in the diagnostic section. Table 4 gives an overview of relevant treatment options.

**Table 4:** Suggested therapeutic methos and clinical indications for upper and lower GIT impairment

Treatment option	Clinical indications
*Oropharyngeal*	
Swallowing therapy performed by Speech and Language therapists	Recommended for PwPs with sufficient cognitive level to follow clinicians’ indications (Schindler et al. 2021)
	Therapy selection should address underlying biomechanical/pathophysiological mechanisms, i.e. (Schindler et al. 2021):
	Thickened liquids to prevent liquid aspiration (Logemann et al. 2008)
	Chin-tuck-maneuver to prevent liquid aspiration (Ayres 2017)
	Training/avoiding dual-task situations (Labeit et al. 2021)
	PD specific swallowing therapy should be preferred (Talebi et al. 2023)
Medical treatment	Levodopa-Responsiveness is supposed to improve the following parameters: pharyngeal residue, penetration, oropharyngeal transit times (Warnecke et al. 2022), (Warnecke et al. 2016)
	Bypassing the GIT (i.e. Apomorphin s.c. or s.l., Transdermal Rotigotine patch) (Hirano et al. 2019; Torti et al. 2019)
	Jejunal and subcutaneous levodopa are able to improve swallowing function as well (Hirano et al. 2025; Labeit et al. 2020, 2025)
	A FEES-Levodopa-Test should be performed to objective a treatment response of Levodopa to OD in PwPs (Warnecke et al. 2016)
*Behavioral swallowing therapy*	
Video-assisted swallowing therapy (VAST^®^)	Visualization of swallowing function might be helpful for providing biofeedback to PWP (Manor et al. 2013)
Expiratory muscle strength training (EMST^®^)	4-week-training period, improvement of swallowing safety and efficacy with suggested detraining effects (Claus et al. 2021; Troche et al. 2010)
Sensorimotor training for airway protection (smTAP)	Improvement of cough reflex and voluntary cough function (Troche et al. 2023)
BiSSkiT Biofeedback in Swallowing Sklill Training	Might produce functional, biomechanical and swallowing-related quality of life improvements (Athukorala et al. 2014)
Lee Silverman Voice treatment (LVST^®^)	Originally developed for treatment of dysarthophonia, but might improve oropharyngeal dysphagia as well (Sharkawi et al. 2002)
*Esophageal*	
Medical treatment	Botulinumtoxin injections in the upper esophageal sphincter (UES)* (Triadafilopoulos et al. 2017)
	Capsaicin might improve esophageal motility and UES contraction * (Suntrup-Krueger et al. 2021)
*Gastroduodenal*	
Medical treatment	Check medication that may further gastric emptying (e.g. pantoprazole, neuroleptics or opioids)
	Introduce domperidone (but might increase risk of long QT syndrome)
	Increase of levodopa dose might worsen delayed gastric emptying
	Nitazidine/ghrelin (experimental) (Warnecke et al. 2022)
*Colorectal*	
Constipation prophylaxis	Sufficient liquid intake (1,5–2 L/d)
	Fiber-rich diet
	Stool softener
	Probiotics and prebiotics
	Regular physical exercise (Leta et al. 2023; Jost 2017)
Medical treatment	Probiotics
	Osmotic laxatives (e.g. Macrogol, Movicol^®^, Lactulose)
	Stimulant laxatives (e.g. Bisacodyl; Sodium picosulfate)
	Prucaloprid (Warnecke et al. 2022; Leta et al. 2023)

*Only occasional cases,* usually performed by gastroenterologists*

With regard to use of aforementioned medication, it should be explicitly noted that any possible contraindications as well as interactions with the patient’s existing medication must be carefully reviewed in each individual case.

For other suggested therapies like neuromuscular electrical stimulation (NMES) or surface electromyography (EMG), recent studies indicate a possible positive effect on PD-related dysphagia (Krasko et al. 2023; Park et al. 2018). However, clinical evidence is inconsistent to support an unequivocal recommendation, and a possible treatment indication should only be determined by appropriate specialists (Warnecke et al. 2022; Krasko et al. 2023).

## Emergency treatment and further specific issues

In case of an acute exacerbation of GIT symptoms in PwP coexisting, medical reasons should be excluded at first as alpha-synuclein pathology is usually not worsening within short periods of time. Concerning oropharyngeal dysphagia, bacterial or viral systemic infections, but also dehydration especially in high outdoor temperatures, or following surgery intervention might worsen patient’s local or general condition culminating to akinetic crisis at worst and therefore secondarily cause an increase of dysphagic symptoms. General neurologists as well as outpatient treatment teams should address these differential diagnoses and provide appropriate diagnostics carefully.

Besides, in rare cases, other severe neurological diseases, might cause similar symptoms as well: in case of acute onset or worsening of dysphagia without any clinical sign of infection, i.e. brainstem stroke in the dorsolateral medualla oblongata should be considered as well, which should be addressed by further diagnostic evaluation immediately. In cases of acute severe constipation or overflow diarrhea, gastrointestinal complications, i.e. an ileus, could be causative and a detailed (instrumental) GIT examination would be indicated by an appropriate specialist.

In these cases, a temporary switch to non-oral therapy could be indicated. Concerning oral dopamine agonist therapy, a change to the transdermal rotigotine patch might be helpful. In addition, several on-demand therapies (such as subcutaneous or sublingual apomorphine or orally inhaled levodopa) as well as non-oral device-aided therapies are able to bypass the GIT (Warnecke et al. 2022; Olanow et al. 2021), even if only temporally until dysphagia improved. Another possibility, at least transitionally, may be rectal administration of levodopa (Guthrie et al. 2025; Vogelzang et al. 2025). Intravenous application of amantadine is also possible but often causes side effects. Concerning intrajejunal or subcutaneous infusion systems, there is growing evidence for a possible improvement of GIT symptoms as well. The use of levodopa-carbidopa ininal gel (LCIG) infusion therapy might be capable of reducing pharyngeal bradykinesia and premature bolus spillage (Krasko et al. 2023; Galli et al. 2025). Preliminary data shows slight effects of using subcutaneous foslevodopa/foscarbidopa infusion therapy as well (Hirano et al. 2025; Labeit et al. 2025). In case of deep brain stimulation (DBS), there is only limited information about GIT affection like oropharyngeal dysphagia or esophageal motility available so far (Warnecke et al. 2022; Krasko et al. 2023). However, in cases of acute worsening of symptoms, DBS should be reassessed by an appropriate specialist.

If severe dysphagia is diagnosed via instrumental tools with significantly increased risk for aspiration events, affected patients and close family members should be informed as soon as possible and interprofessional discussion of further treatment options should be provided including i.e. SLPs, gastroenterologists, clinical nutritionists and general practitioners. Depending on evaluation resulting potential risks, changes in medical applications or modified diet texture as well as the option of a PEG (feeding tube) with all resulting consequences should be considered. Besides, continued treatment of behavioral interventions might not be appropriate in these cases depending on sufficient level of cognitive function to follow clinicians’ indications in the affected PwPs as well (Schindler et al. 2021).

## Treatment pathways and Follow-up recommendations

As mentioned above, due to the authors’ clinical experience, key questions about GIT symptoms should be asked every 3–6 month in clinical setting, especially in patients with the “body-first” subtype. Depending on answers as well as clinical impression and/or findings at physical examination, further screening methods as well as instrumental examination should be performed at different GIT levels depending on the most affected. Figure 2 gives an overview about possible diagnostic pathways. When instrumental tools reveal abnormal findings, treatment should start irrespective of the stage of the disease (Labeit et al. 2022; Schindler et al. 2021). Depending on severity of evaluated symptoms, follow-up examinations should be performed every 12 months to evaluate the effect of therapy due to the authors’ clinical experience. In case of mild affection, follow-up examinations should be performed every 24 months. In patients with severe affection, i.e. the presence of aspiration events, follow-up after 6 months should be preferred.

**Fig. 1:**
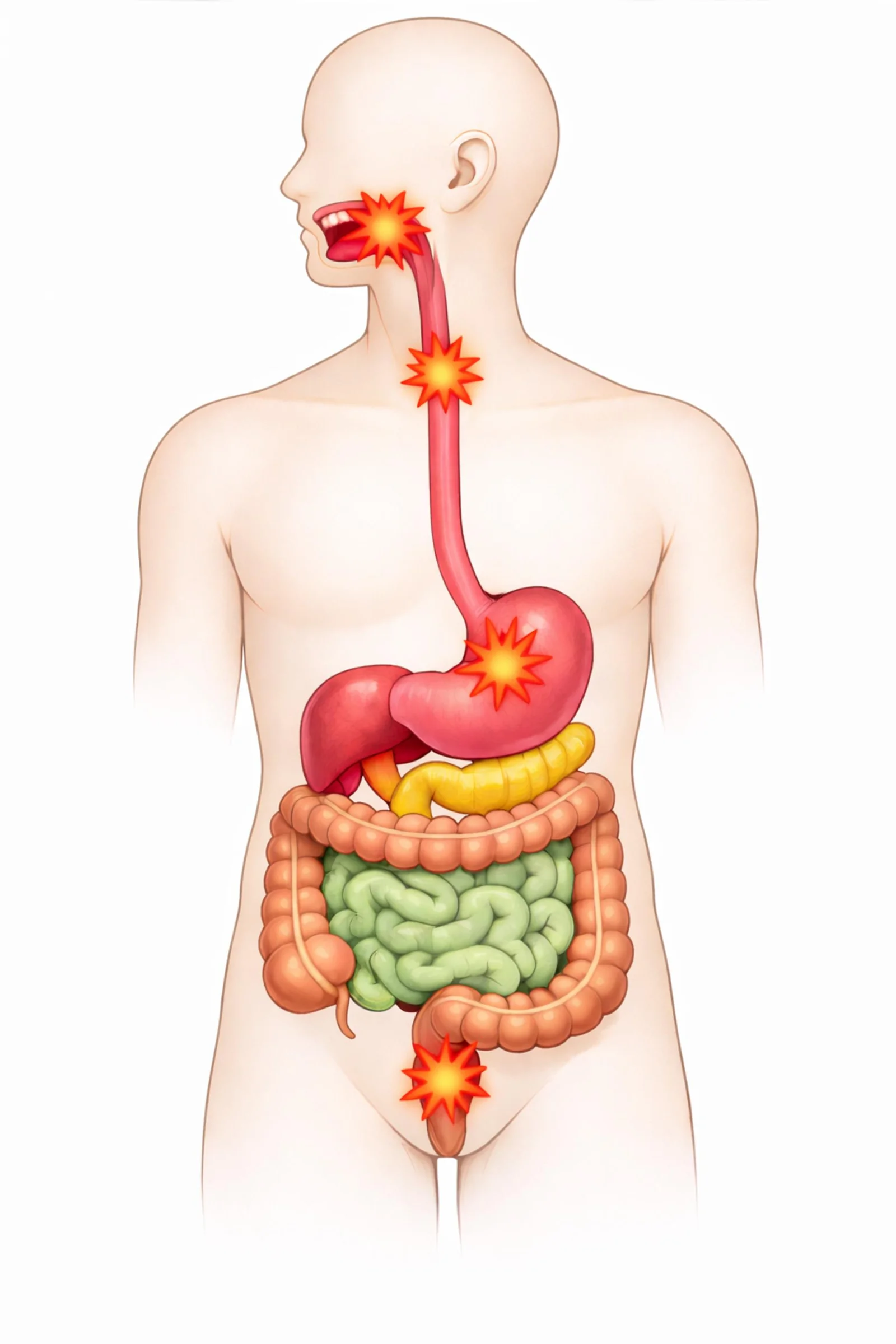
Schematic figure of the GIT in PwPs, showing typical locations of most pronounced impairment. For possible questions to be addressed see Table 2

**Fig. 2:**
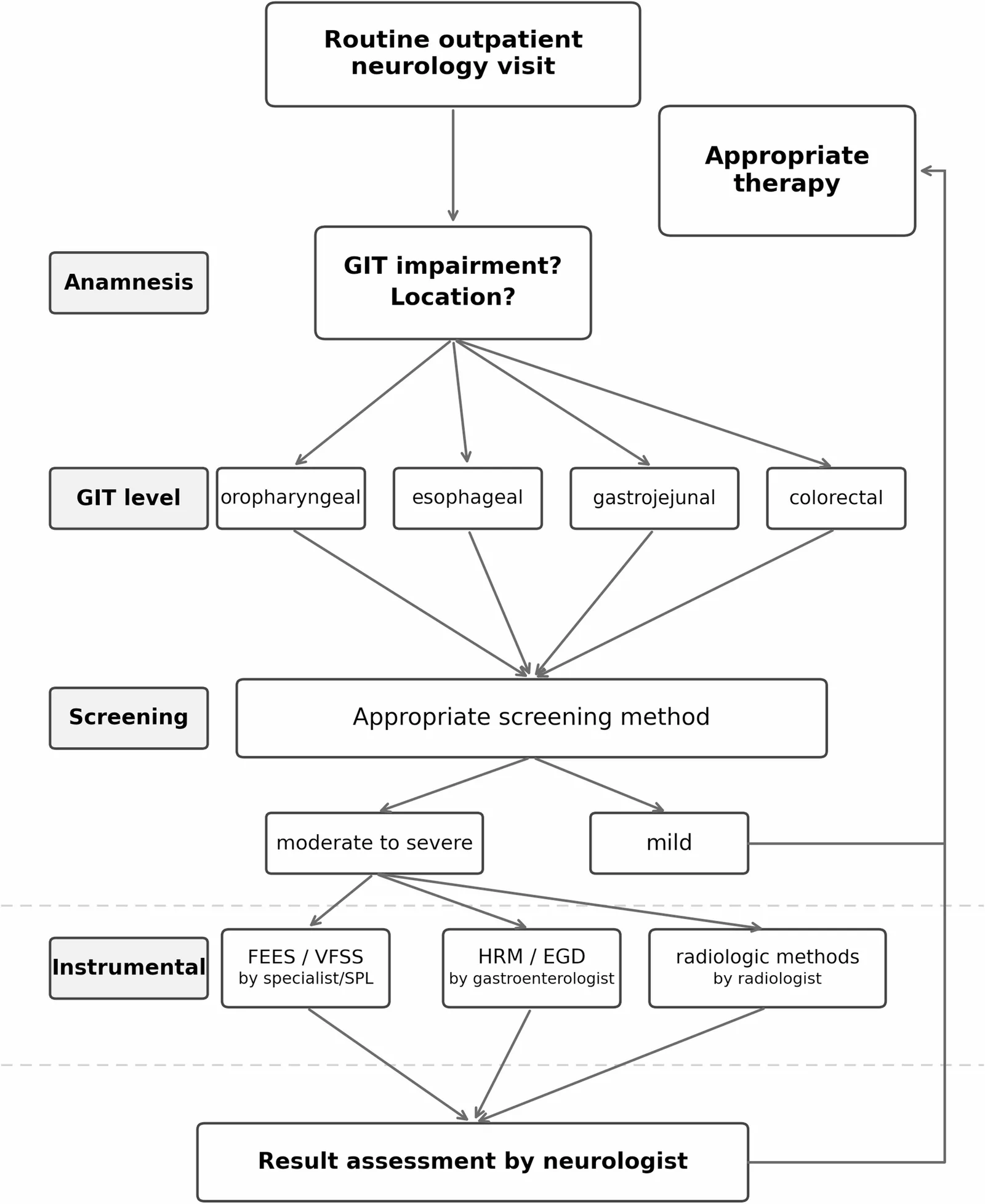
Suggested pathway for PD outpatient care teams for sufficient identification of GIT impairment level, selection of appropriate screening methods and – if necessary – further instrumental diagnostic tools performed by specialists to initiate effective treatment

## Quality assurance

In order to keep the medical knowledge provided up to date and herewith to ensure the quality of the SOP, we recommend an annual review and revision if necessary.

## Discussion

The suggested SOP based on expert consensus proposals was developed during a workshop held as part of a German nationwide PD expert meeting. The SOP suggestions are based to a large extent of clinical experience in examination, diagnosis and treatment of GIT disturbance in PwP provided by the participating authors (Suttrup and Warnecke 2016; Warnecke et al. 2022; Buhmann et al. 2019).

In lively discussions, it quickly became clear that, in our point of view, that the first step in detecting gastroininal disorders is to take an adequate medical history and identify possible GIT abnormalities. Outpatient care teams should initially use clear, simple questions to screen for potential GIT problems and, if patients report any impairment, initiate further evaluation by using available screening methods, i.e. validated questionnaires or other screening tools (Suttrup and Warnecke 2016; Warnecke et al. 2022; Leta et al. 2023). If mild GIT affection is supposed, timely treatment initiation should be arranged, i.e. by prescribing speech and language therapy in oropharyngeal dysphagia. In more severe cases, further instrumental evaluation by appropriate specialists should be promoted. Once the examination results are available, the outpatient care team can select the treatment which is needed to address specific GIT impairment patterns, if necessary, supported by the specialists’ expertise.

During discussion of potential screening methods, it became clear that not all recommended questionnaires or clinical tests in the field of PD have sufficient reliability to ensure detection of all types of GIT disorders. However, since instrumental diagnostic evaluation cannot be performed immediately in every patient for health economical as well as logistical reasons alone, every available screening tool should be used to gather more information about possible dysfunction patterns. In this way the outpatient team might better appraise whether further diagnostic specialized evaluation is necessary and appropriate further assessment can be initiated.

The need for reliable detection of gastrointestinal emergencies that may require immediate further treatment in a specialized center equally deserves active surveillance. Here, the outpatient care team is responsible for recognizing severe GIT disturbances and to take treatment decisions for the affected patients without delay.

## Conclusion

In this manuscript, we suggest a detailed SOP addressing general neurologists as well as other members of interprofessional neurological outpatients care or MD specialist teams to provide structured procedural guidance for adequate anamnesis, diagnosis, treatment options and follow-up monitoring of swallowing disorders as well as other gastrointestinal impairment in PwP.

## Data Availability

No datasets were generated or analysed during the current study.

## Abbreviations

CCS: Cleveland constipation scale
DBS: Deep brain stimulation
DGE: Delayed gastric emptying
EGD: Esophagogastroduodenoscopy
EGG: Electrogastrography
EMG: Electromyography
EMST®: Expiratory muscle strength training
FEES: Fiberoptic endoscopic evaluation of swallowing
GDS: Gut dysmotility questionnaire
GIT: Gastrointestinal tract
H&Y: Hoehn and Yahr
HP: Helicobacter pylori
HRM: High resolution manometry
LVST®: Lee silverman voice treatment
MDS-UPDRS: Movement disorders society-unified Parkinson’s disease rating scale
MRI: Magnetic resonance imaging
NMES: Neuromuscular electrical stimulation
NMSQ: Non-motor symptom questionnaire
OD: Oropharyngeal dysphagia
PAC-SYM: Patient assessment of constipation-symptoms
PD-NMS: Non-motor symptoms questionnaire
PD: Parkinson’s disease
PEG: Percutaneous endoscopic gastrostomy
PwP: People with Parkinson’s disease
SCOPA-AUT: Scales for outcomes in Parkinson’s disease – autonomic dysfunction
SIBO: Small intestinal bacterial overgrowth
SLP: Speech and language therapist
smTAP: Sensorimotor training for airway protection
SOP: Standard operating procedure
STN: Subthalamic nucleus
Tc: Technetium
VAST: Video-assisted swallowing therapy
VFSS: Videofluoroscopy
